# Comparative efficacy of fascia lata allograft, platelet-rich fibrin, and connective tissue graft for peri-implant soft tissue augmentation: a randomized controlled clinical trial

**DOI:** 10.1186/s12903-026-09589-5

**Published:** 2026-08-20

**Authors:** Ahmed Mohamed Elhadidy, Hala H Hazzaa, Ahmed Mohamed Ali Elgendy, Enas Ahmed Elgendy

**Affiliations:** 1 Department of Oral Medicine, Periodontology, Diagnosis and Oral Radiology, Delta University for Science and Technology, Gamasa, Egypt; 2 Department of Oral Medicine, Periodontology, Diagnosis and Oral Radiology, Faculty of Oral and Dental Medicine for girls, Al Azhar University, Cairo, Egypt; 3 Department of Oral Medicine, Periodontology, Diagnosis and Oral Radiology, Faculty of Oral and Dental Medicine, Kafrelsheikh University, Kafrelsheikh, Egypt

**Keywords:** Dental implants, Gingival phenotype, Soft tissue augmentation, Connective tissue graft, Platelet-rich fibrin, Fascia lata allograft

## Abstract

**Background:**

Adequate peri-implant soft tissue thickness and keratinized mucosa width are crucial for implant health and aesthetics. Autogenous subepithelial connective tissue grafts (CTG) are the gold-standard for augmenting thin biotypes, but they require a second surgical site. This trial evaluated whether a human fascia lata allograft (FLA) or platelet-rich fibrin (PRF) membrane could comparably enhance peri-implant soft tissue phenotype and serve as less invasive alternatives of CTG.

**Methods:**

Forty-five patients with a thin gingival phenotype (≤ 1 mm thickness) requiring upper anterior implant were randomly assigned to receive soft tissue augmentation with either (1) FLA allograft, (2) PRF membrane, or (3) CTG (control). Interventions were applied concurrently with implant placement in a standardized manner. Gingival thickness (GT) and keratinized tissue width (KTW) were measured at baseline and 6 months using transgingival probing and clinical indices. Labial bone thickness was assessed by cone-beam CT at implant placement and 6 months.

**Results:**

Forty-five enrolled patients completed the study. All treatment modalities showed improvements in gingival and bone parameters, with the connective tissue graft consistently demonstrating the greatest gains. The median keratinized tissue width increased to 4.00 mm in the Connective Tissue Graft group, slightly higher than the Fascial Lata Allograft and Platelet-Rich Fibrin groups (3.75 mm). Gingival thickness showed significant increases in all groups, with the Connective Tissue Graft reaching 2.15 mm, followed by Fascia Lata Allograft (1.80 mm) and Platelet-Rich Fibrin (1.55 mm). Labial bone thickness also increased, with the Connective Tissue Graft achieving the highest mean value of 2.300 mm, and after 6 months, significant gains were observed in both this group (*p* = 0.032) and in the Platelet-Rich Fibrin group (*p* = 0.044), while Fascia Lata Allograft showed a smaller, non-significant increase.

**Conclusions:**

Both fascia lata allograft (FLA) and PRF improved peri-implant soft tissue parameters, however the connective tissue graft (CTG) remains the gold standard for achieving the greatest thickness and best aesthetic results. FLA is a promising alternative that avoids a second surgical site with comparable outcomes, whereas PRF offers faster healing but lacks the volume necessary for significant tissue modification.

## Introduction

“Peri-implant phenotype” is a combination of soft tissue thickness (gingival biotype), keratinized mucosa width, the supracrustal tissue height, and peri-implant bone thickness. These factors have been strictly recognized as a critical determinant of implant success [1]. A thick peri-implant mucosa (> 2 mm) can better resist inflammatory breakdown and is linked to more stable marginal bone levels and superior aesthetic outcomes. Consequently, enhancing the peri-implant soft tissue volume and keratinized tissue has become an integral step in implant therapy, especially in the esthetic zone [2].

Different surgical approaches have been encountered to augment thin gingival phenotypes around implants. The subepithelial connective tissue graft (CTG) harvested from the palate remains regarded as the reference standard for soft tissue augmentation due to its predictable, long-term volume stability. CTG procedures increase mucosal thickness and can improve peri-implant indices and esthetic scores, as the autograft’s live fibroblasts and matrix promote strong tissue integration [3].

However, CTG harvest entails a second surgical site, which adds patient morbidity (donor site pain, bleeding) and has limited tissue availability. These drawbacks have prompted exploration of substitutes such as acellular dermal matrices and other allografts or xenografts that eliminate donor harvesting. Indeed, allogeneic acellular dermal matrix has shown comparable outcomes to CTG in some studies, with similar gains in thickness and keratinized mucosa width, while significantly reducing patient discomfort [4–6].

Human fascia lata allograft (FLA) is a recently introduced soft tissue substitute derived from the dense connective tissue of the lateral thigh. Processed Fascia lata membranes are acellular collagenous matrices, supplied sterile for clinical use. The fascia lata graft offers a long, durable collagen scaffold that can be adapted to the implant site, functioning analogously to autogenous CTG [7]. Furthermore, the graft is clinically safe, as it does not trigger a significant immunological foreign body response.

A key advantage of fascia lata is the elimination of donor site morbidity while retaining structural integrity for cell repopulation [8]. It serves as a highly effective clinical alternative for correcting Seibert type 1 ridge defects, offering growth characteristics comparable to connective tissue. This material is particularly advantageous in periodontics because it exhibits superior handling properties with pontics, which facilitates greater papilla filling. Finally, its use provides significant surgical flexibility by overcoming the anatomical thickness and size limitations inherent to traditional palate donor sites [9].

Another strategy to enhance peri-implant soft tissues without a secondary surgery is the use of platelet-rich fibrin (PRF), an autologous blood concentrate. PRF consists of a fibrin matrix containing platelets and bioactive growth factors (e.g. PDGF, TGF-β1, VEGF) that are gradually released to stimulate angiogenesis and fibroblast proliferation [10]. Compared to CTG or allografts, PRF provides biological stimulation rather than bulk volume. Clinically, PRF has been applied as a membrane interposed under flaps or around implants to promote soft tissue healing and modestly increase thickness [11].

Recently studies carried out by Miron et al., concluded that PRF can effectively increase keratinized mucosa width and soft tissue thickness around implants, of low cost, ease of procurement, and minimal patient morbidity though the gains are often smaller than those achieved with connective tissue grafts [12, 13].

Limited evidence is currently available regarding the efficacy of FLA relative to PRF and the CTG as gold-standard in enhancing the peri-implant soft tissue phenotype. Therefore, this randomized controlled trial was conducted to compare periodontal phenotype (thickness and width of keratinized tissue, labial bone thickness) around dental implant in upper anterior region across three approaches in patients with a thin phenotype. The null hypothesis was there would be no significant difference in keratinized tissue width and labial bone level between (FLA and PRF) relative to the gold-standard CTG.

## Materials and methods

### Study design and setting

This study was designed as a three-arm, parallel-group, open label, randomized controlled clinical trial and is reported in accordance with the CONSORT 2010 guidelines. The trial was conducted at the Department of Periodontology, Kafrelsheikh University (Egypt), after approval of the protocol by the University Research Ethics Committee received (ethical approval number (KFSIRB200-87) on 25/12/2023). All participants provided written informed consent. The study was registered on (http://www.clinicaltrials.gov: (NCT06219473) on 12 of January 2024) prior to patient enrollment and the date of study start was 1/2/2024 and study completion was 01/02/2025)

Sample size was calculated using the G*power, version 3.1.9.2, Universität Kiel, Germany based on comparing the gingival thickness (GT) six months after implant placement among patients treated with SCTG and PRF [14].

Mean ± SD of GT among patients treated with SCTG = 3.0 ± 0.7.

Mean ± SD of T1 among patients treated with PRF = 2.13 ± 0.46.

α error probability = 0.05.

Power (1-β error probability) = 0.90.

So, the minimum sample size for each group will be 11 patients. And adding 20% dropout rate.

Totally 45 patients. Allocated equally into three groups, 15 patients treated with sub-epithelial connective tissue graft (SCTG), another 15 patients treated with platelet-rich fibrin (PRF)graft, and another 15 patients treated with Fascia Lata Allograft.

#### Eligibility criteria

Participants were adults with ages ranged of 20–45 years old, systemically healthy (ASA I), with a missing maxillary anterior tooth (incisor, canine) indicated for implant placement, presenting a thin gingival biotype (≤ 1 mm gingival thickness by probe transparency) and ≥ 2 mm of pre-existing keratinized gingiva at the site. Sufficient alveolar bone volume to place a standard implant without bone grafting was required (intact labial plate on CBCT). Patients had to demonstrate good oral hygiene and compliance.

Smokers, tobacco users, pregnant females, patient with bruxism or occlusal dysfunction and active periodontal disease/ poor oral hygiene were excluded from the trial.

### Randomization

**Fig. 1:**
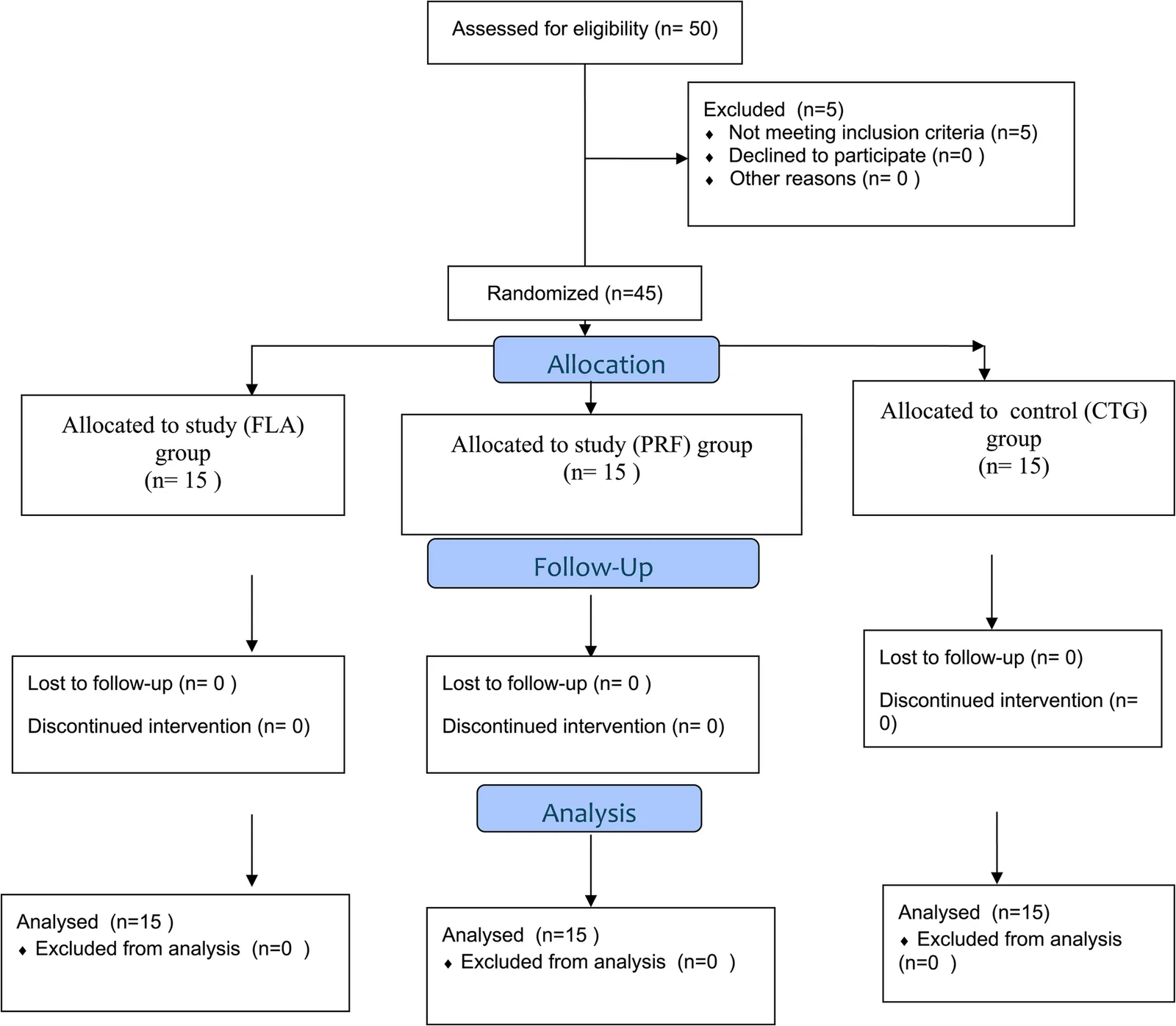
Consort flow chart showing study design

Patients were randomly assigned (allocation ratio 1:1:1) to one of three intervention groups: Group 1: Fascia Lata Allograft (FLA); Group 2: Platelet-Rich Fibrin (PRF); Group 3: Subepithelial Connective Tissue Graft (CTG). A computer-generated random sequence (block size 5) was prepared by an independent statistician. Concealed allocation was achieved using sequentially numbered opaque envelopes opened on the day of surgery (Fig 1).

### Surgical procedures

All surgical procedures were performed by a single experienced periodontist to ensure standardization and minimize operator-related variability. For all groups, the implant placement and soft tissue augmentation were performed in a one-stage surgical procedure under local anesthesia. Pre-surgical prophylaxis (0.2% chlorhexidine rinse and antibiotic as indicated) was given. A mid-crestal incision with intrasulcular extensions was made in the edentulous site, and a full-thickness mucoperiosteal flap was elevated labially. After minimal flap reflection, a dental implant (of suitable diameter and length for the site) was placed in the prepared osteotomy following the manufacturer’s protocol, achieving good primary stability.

**Fig. 2:**
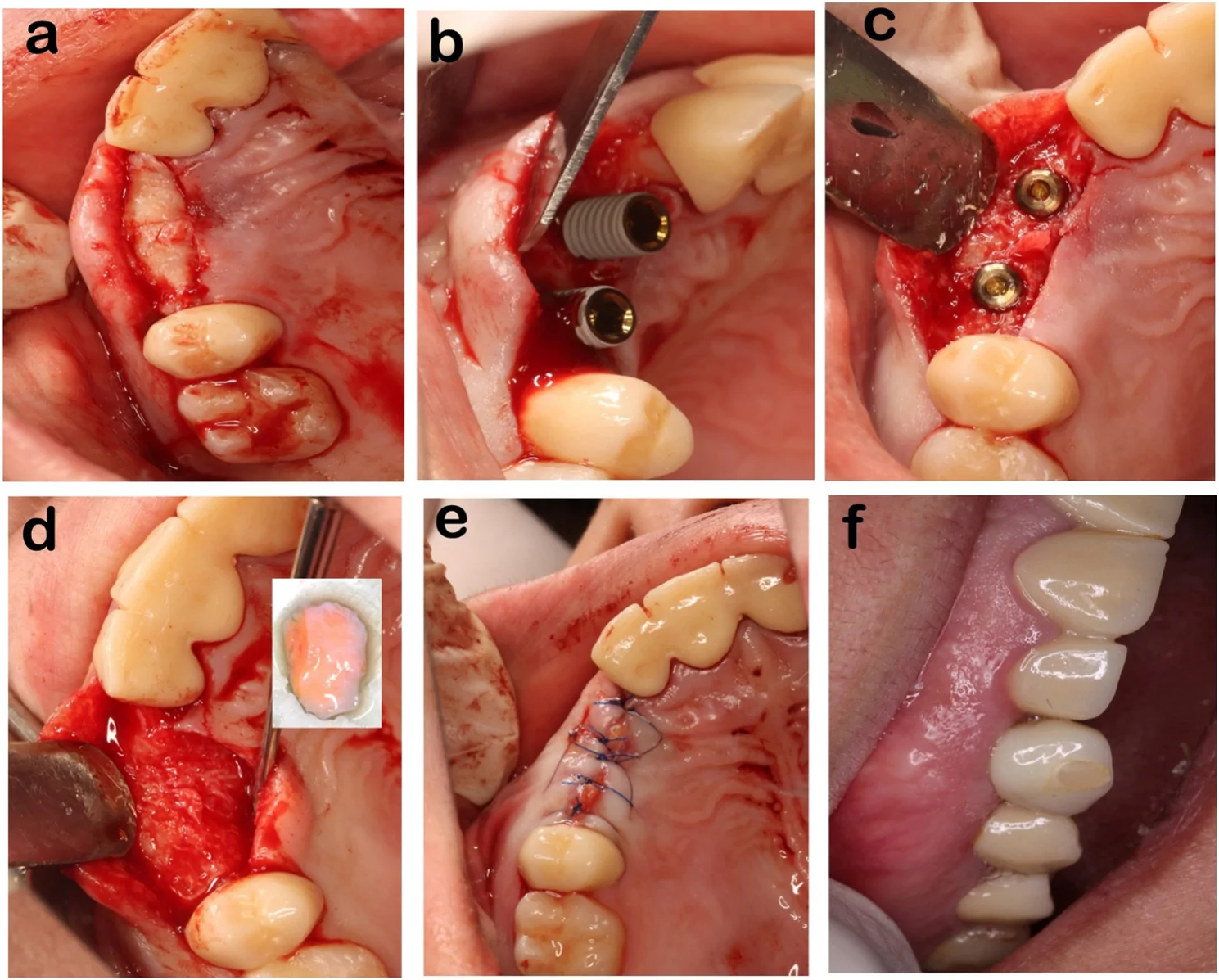
**a** A crestal incision was given in the edentulous area. **b**-**c** Full- thickness flap elevation and implant placement in upper right canine (**d**) Connective tissue graft harvested from patient palate and positioned under the labial flap. **e** Wound closure and suturing. **f** Final restoration at 6 months

**Fig. 3:**
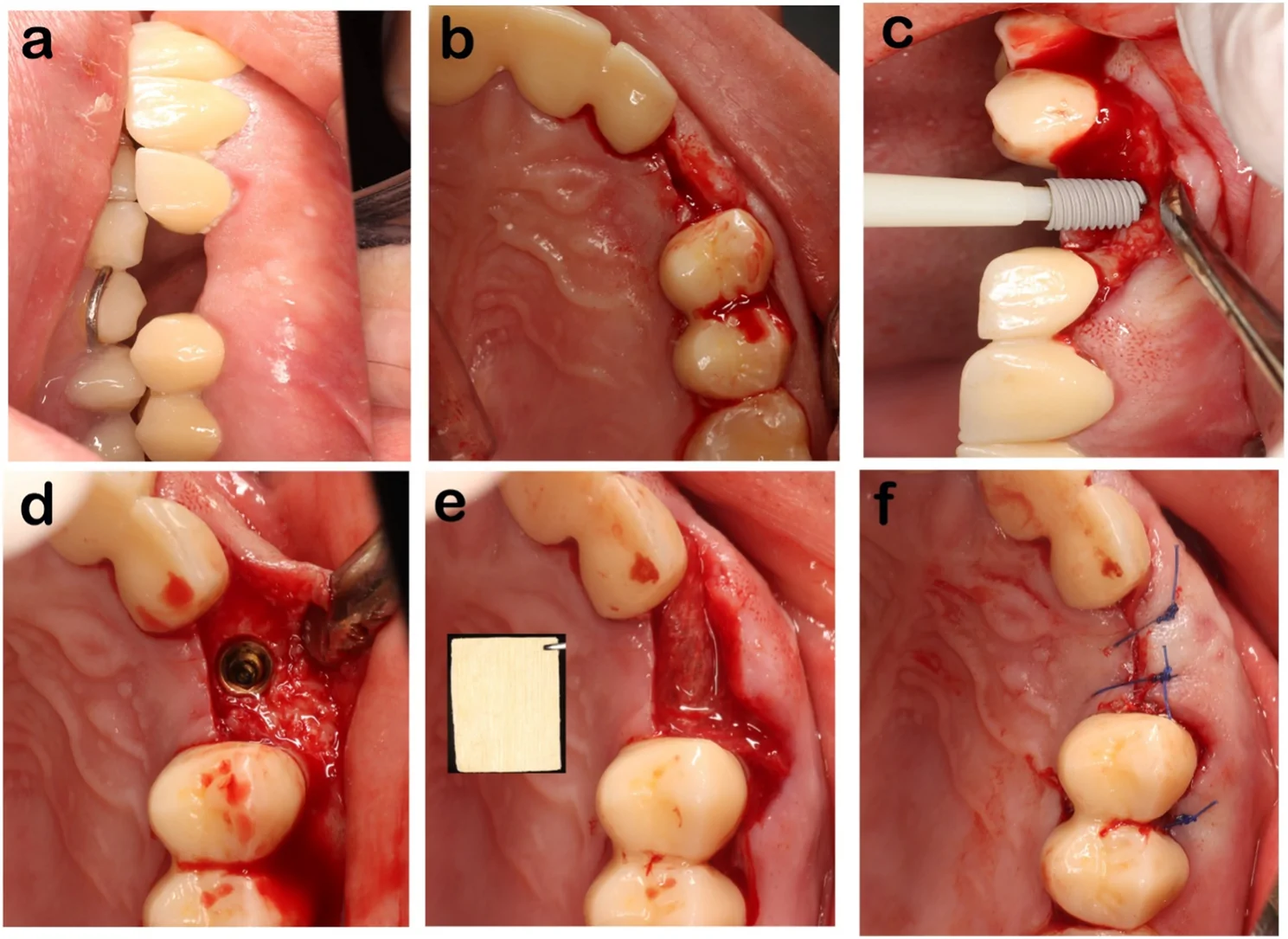
**a** Preoperative view of edentulous upper left canine area. **b** A crestal incision was given in the edentulous area. **c**-**d** Full- thickness flap elevation and implant placement in upper left canine. **e** Fascia Lata positioned under the labial flap. **f** Wound closure and suturing

**Fig. 4:**
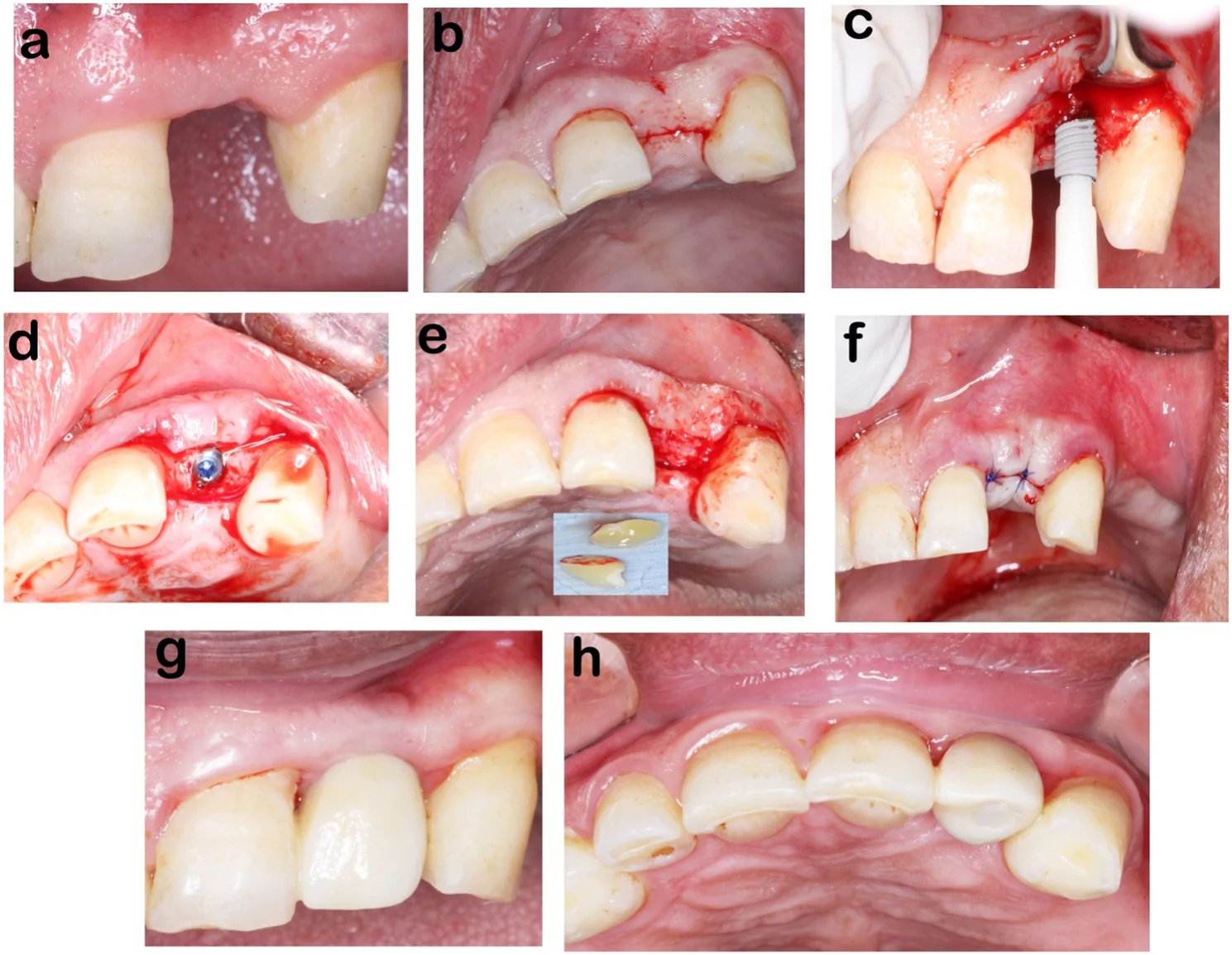
**a** Preoperative view of edentulous upper lateral incisor area. **b** A crestal incision was given in the edentulous area. **c**-**d** Full- thickness flap elevation and implant placement in upper lateral incisor. **e** PRF membrane positioned under the labial flap. **f** Wound closure and suturing. **g**-**h** Final restoration after 6 months

- Connective Tissue Graft group (CTG): A subepithelial connective tissue graft was harvested from the patient’s hard palate. A single-incision technique was used to obtain a graft approximately 1.5 × 1.0 cm in size and 1–1.5 mm thick. The CTG was placed onto the labial aspect of the implant site: it was positioned over the buccal peri-implant area to augment the soft tissue thickness. The graft was sutured in place if needed with resorbable sutures. Donor site palatal wound was closed with sutures and protected with a periodontal dressing (Fig. 2) [15].
- Fascia Lata Allograft group (FLA): A dehydrated fascia lata allograft membrane (Fasia lata allograft from Maxxeus company) was rehydrated in sterile saline per manufacturer instructions. A section of the membrane (approximately matching the defect dimensions, e.g. ~1.5 × 1.0 cm) was trimmed and placed on the labial aspect around the implant. Similar to the CTG, the fascia graft was adapted to cover the buccal ridge contour and tucked under the labial flap, aiming to increase the soft tissue bulk. The membrane was secured under the flap without additional pinning (the flap closure holds it in place). (Fig. 3) [16].
- Platelet-Rich Fibrin group (PRF): Prior to surgery, intravenous blood (approximately 20–30 mL) was drawn from the patient into 10 mL sterile glass tubes without anticoagulant. The tubes were immediately centrifuged at 3000 rpm for 10 min and processed to prepare (L-PRF) following the standard protocol by Choukroun et al. The blood was centrifuged immediately to obtain PRF clots, which were then compressed into membrane form. Two PRF membranes were layered over the buccal aspect of the implant site under the flap to biologically stimulate soft tissue healing and potentially add a slight fibrin volume. PRF membranes (≈ 1 mm thick each) were placed to cover the buccal peri-implant area and extend 2–3 mm beyond the implant site margins. They were held in situ by passive flap pressure (no sutures in the membrane) [17]. (Fig. 4)

All surgeries were performed by the same periodontist to minimize technique variability. In all groups, efforts were made to achieve passive, primary closure of the flap to maximize graft take and uneventful healing.

#### Post-operative care

Identical post-operative regimens were followed. Patients were instructed to rinse twice daily with 0.2% chlorhexidine mouthwash for 2 weeks. They received oral antibiotics (amoxicillin 500 mg three times daily for 7 days) and analgesics (ibuprofen 600 mg as needed). Patients were advised to avoid mechanical brushing of the surgical area or undue trauma for 2 weeks. Sutures were removed after 14 days (palatal donor site and any non-resorbable sutures). Professional cleaning of adjacent teeth was done at 1 and 3 months post-surgery. All patients were seen at 1 week, 2 weeks, 1 month, 3 months, and 6 months post-operatively for evaluation and oral hygiene reinforcement.

### Prosthetic phase

At 6 months after implant placement, a second-stage surgery was performed for all implants that had been submerged by using healing abutments. Two weeks later, impressions were taken and definitive single crowns were fabricated. All implants were restored with screw-retained all-ceramic crowns by 6 months post-placement.

### Outcome measures

**Fig. 5:**
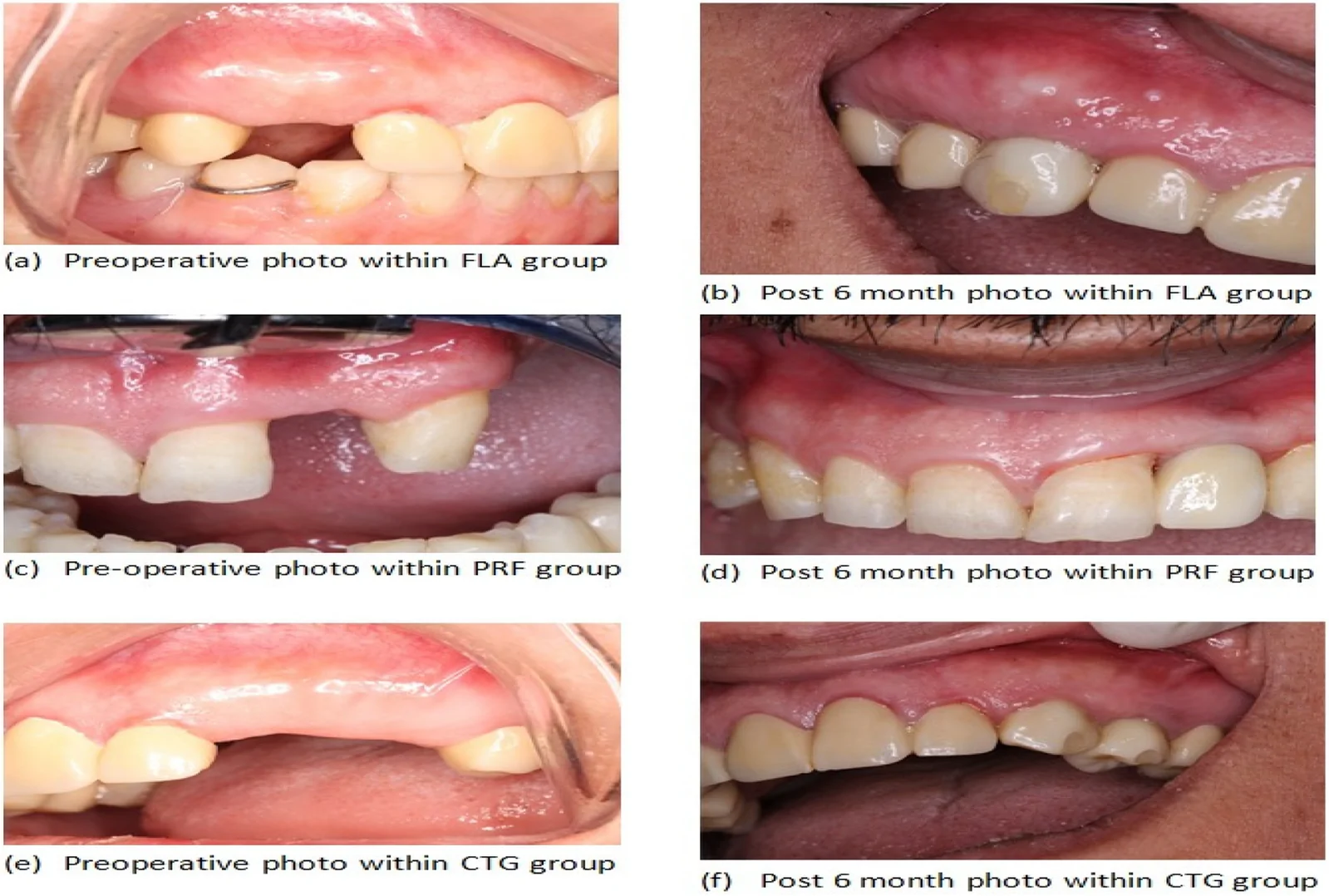
Pre and post operative photos for each group to compare soft tissue contour and aesthetic outcomes

**Fig. 6:**
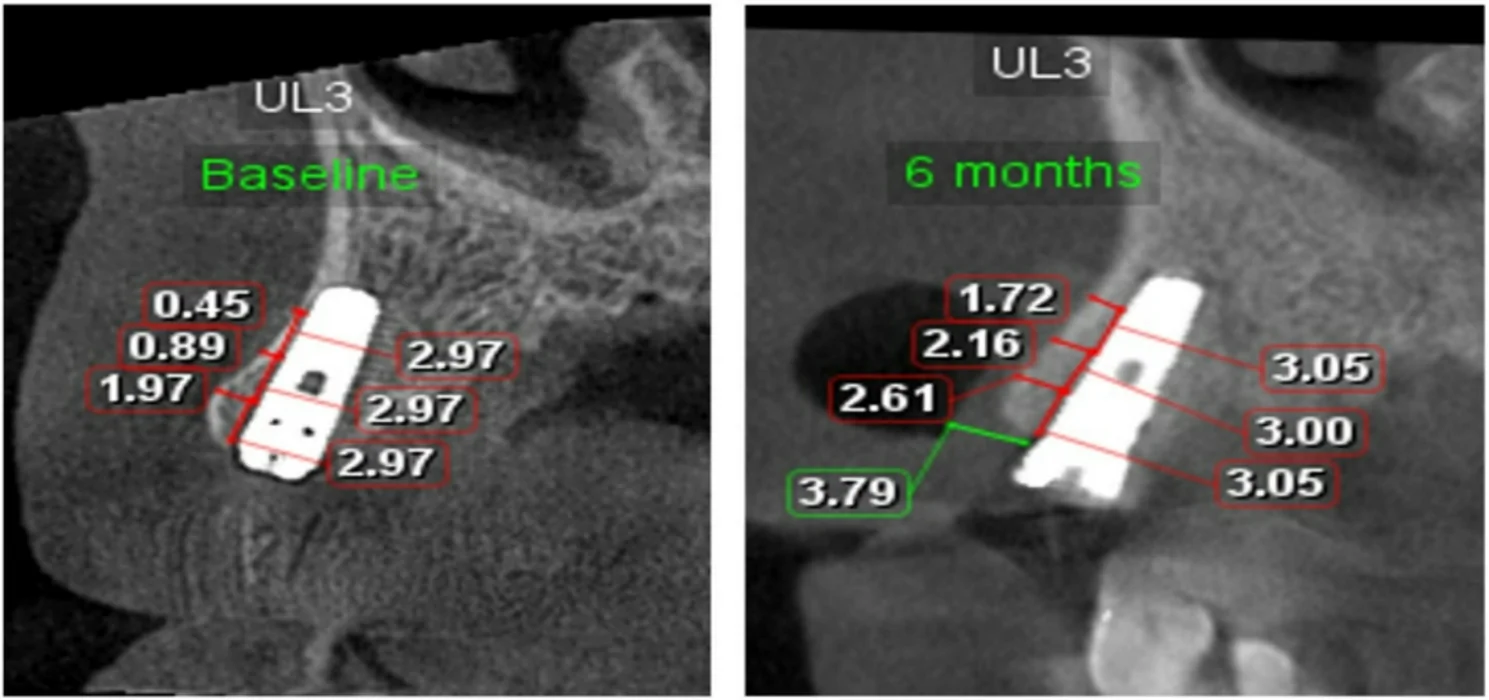
CBCT evaluation of labial bone thickness at baseline and 6 months following connective tissue graft augmentation

**Fig. 7:**
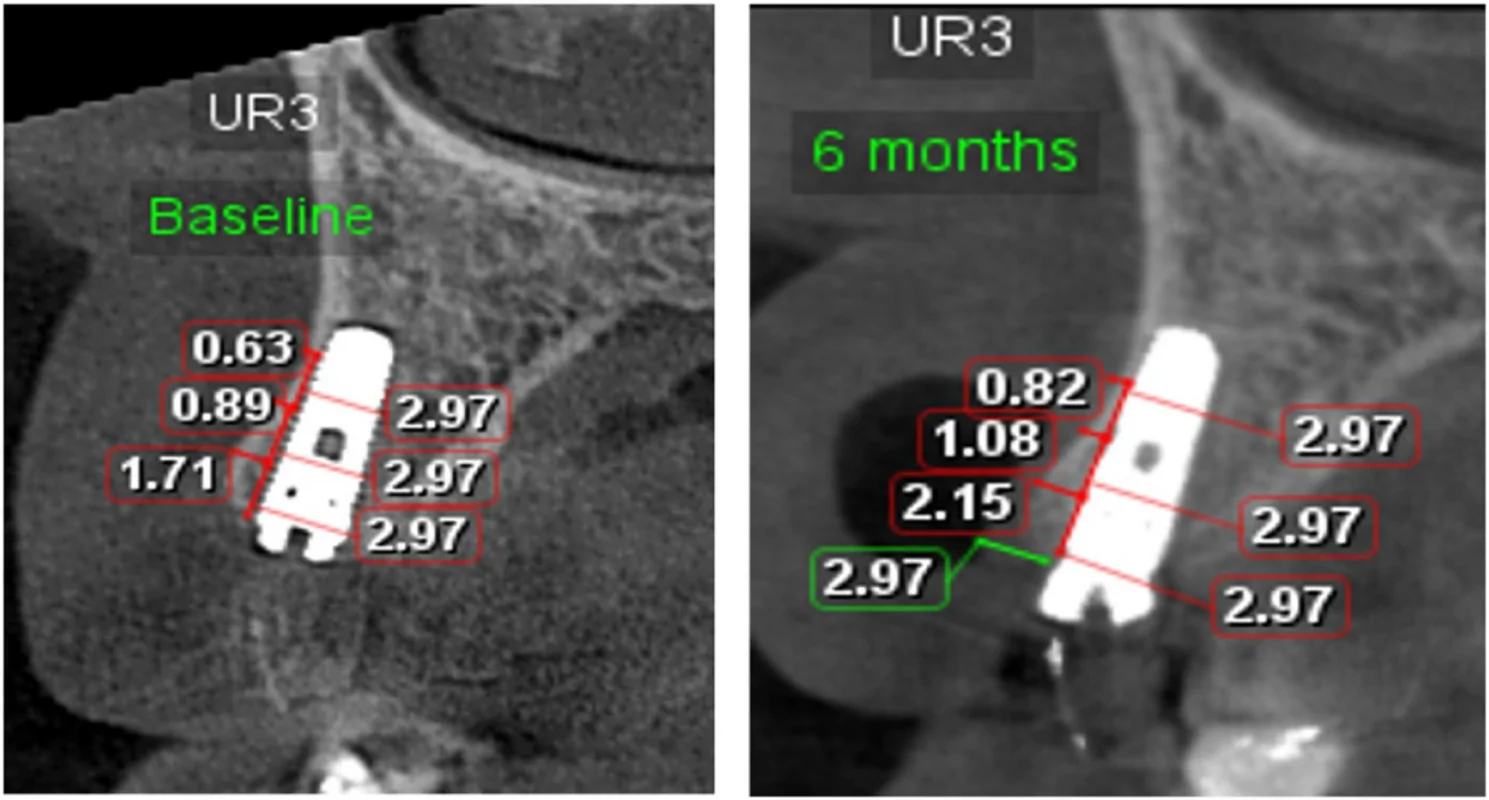
CBCT evaluation of labial bone thickness at baseline and 6 months following Fascia lata membrane

All clinical measurements were performed by a blinded examiner not involved in the surgeries. The following outcomes were recorded:

- Gingival Thickness (GT): Assessed by transgingival probing under local anesthesia using periodontal probe. At the mid-facial aspect of the implant, 2 mm apical to the free gingival margin, the probe was inserted perpendicular to the mucosa until bone contact. Gingival thickness measurements were performed using a standardized periodontal probe (UNC-15) with markings at 1 mm intervals. The penetration depth (soft tissue thickness) was measured to the nearest 0.1 mm (using a digital caliper on the probe). Measurements were taken at baseline preoperative and 6 months post-surgery. Baseline GT confirmed the “thin” phenotype (all ≤ 1 mm). At 6 months, GT reflected the outcome of tissue thickening. (Fig. 5)
- Keratinized Tissue Width (KTW): The width of keratinized mucosa on the facial aspect was measured with a periodontal probe as the distance from the free gingival margin to the mucogingival junction (MGJ) at the mid-point of the implant site. This was done at baseline and 6 months. The MGJ was identified visually (by color/texture change) and with the roll technique.
- Labial bone thickness (LBT): Labial bone thickness was measured on CBCT cross-sectional images at three standardized reference levels (3 mm, 6 mm, and 9 mm apical to the implant platform). Measurements were performed by drawing a tangent line from the outer surface of the labial cortical plate to the implant surface and recorded in millimeters. This was done at baseline and 6 months. Figures 6 and 7 [18, 19].

### Statistical analysis

Data were collected, coded, and analyzed using SPSS version 27. Data normality was assessed using the Shapiro–Wilk test. Continuous data were presented as mean ± standard deviation (SD) if normally distributed and as median (minimum to maximum) if not normally distributed.

Comparing mean across three study groups was tested using One-Way ANOVA test followed by post hoc LSD method. For not normally distributed data, association across three study groups was tested using Kruskal-Wallis test followed by several Mann-Whitney tests and Within-group comparisons were performed using Wilcoxon Signed Ranks test.

Qualitative data were described using numbers and percentages. Association between categorical variables was tested using Fisher’s Exact test as at least 25% of cells had expected counts below 5. The level of significance was considered at p-value < 0.05.

No interim analysis was performed, and no stopping guidelines were applied due to the fixed sample size and short follow-up period of the study.

## Results

### Patient characteristics and surgical details

A total of 45 patients were randomized into three study groups (Fascial Lata Allograft, Platelet-Rich Fibrin, and Connective Tissue Graft groups). Table 1 showed that there were no significant differences in mean age or gender distribution across groups.

**Table 1:** Baseline demographic characteristics

	Fascia Lata Allograft	Platelet Rich Fibrin	Connective Tissue Graft	Test of significance	*p*-value
Sex					
N (%)					
Male	9 (60.0)	6 (40.0)	6 (40.0)	χ^2^ = 1.607	0.448
Female	6 (40.0)	9 (60.0)	9 (60.0)		
Age					
Mean ± SD	30.8 ± 3.299	30.5 ± 3.962	28.2 ± 4.263	F = 0.057	0.140

*F* one-way ANOVA, *FET* Fischer Exact test

Regarding the keratinized tissue width, Table 2 summarized that, at 6 months, the Connective Tissue Graft group demonstrated a slightly higher median keratinized tissue width (4.00 mm) compared to the Fascial Lata Allograft and Platelet-Rich Fibrin groups (both 3.75 mm). This indicates that the Connective Tissue Graft achieved the greatest increase in keratinized tissue width among the three treatment modalities.

**Table 2:** Comparison keratinized tissue width, thickness, and labial bone thickness between different study groups and per each group

		Fascia Lata Allograft	Platelet Rich Fibrin	Connective Tissue Graft	Test of significance	*p-value*
keratinized tissue width	**Baseline**					
	Median (Min. to Max.)	3.75 (3.50 to 4.00)	3.75 (3.50 to 4.00)	3.75 (3.50 to 4.00)	H = 0.00	> 0.999
	**After 6 months**					
	Median (Min. to Max.)	3.75 (3.50 to 4.37)	3.75 (3.50 to 4.00)	4.00 (3.50 to 4.00)	H = 0.817	0.664
	*p*-value ^#^	0.083	> 0.999	0.083		
Gingival thickness	**Baseline**					
	Median (Min. to Max.)	1.00 (0.90 to 1.38)	1.00 (0.90 to 1.15)	1.00 (0.83 to 1.00)	H = 0.500	0.779
	**After 6 months**					
	Median (Min. to Max.)	1.80 (1.70 to 2.30)	1.55 (1.50 to 1.90)	2.15 (1.80 to 2.45)	H = 6.932	**0.031**; *p*1 = **0.030** *p*2 = 0.366 *p*3 = **0.026**
	*p*-value ^#^	**0.011**	**0.011**	**0.012**		
Labial bone thickness	**Baseline**					
	(Mean ± SD)	1.600 ± 0.120	1.613 ± 0.164	1.763 ± 0.160	F = 2.939	0.075
	**After 6 months**					
	(Mean ± SD)	2.238 ± 0.119	2.150 ± 0.151	2.300 ± 0.131	F = 2.518	0.105
	*p*-value ^$^	0.323	**0.044**	**0.032**		

^#^ : Wilcoxon Signed Ranks test, H: Kruskal Wallis test followed by several Mann-Whitney tests *p*1: between FLA and PRF *p*2: FLA and CTG *p*3: between PRF and CTG; ^$^ :Paired sample t test, F: one-way ANOVA followed by post hoc LSD method

In addition, within group changes from baseline to 6 months, the Connective Tissue Graft group showed a noticeable increase in keratinized tissue width from 3.75 mm to 4.00 mm, whereas the Fascial Lata Allograft and Platelet-Rich Fibrin groups maintained a median width of 3.75 mm. Despite minor clinical shifts in median values, none of the three modalities achieved a statistically significant expansion of keratinized tissue width at the 6-month follow-up.

Regarding gingival thickness, Table 2 viewed that after 6 months, the Connective Tissue Graft group showed the greatest increase in median gingival thickness (2.15 mm), followed by the Fascial Lata Allograft group (1.80 mm) and the Platelet-Rich Fibrin group (1.55 mm). Statistical analysis revealed a significant difference between the groups (H = 6.932, *p* = 0.031), with post-hoc comparisons showing that the Connective Tissue Graft group achieved significantly higher gingival thickness than the Platelet-Rich Fibrin group (*p* = 0.026), while the difference between Fascia Lata Allograft and the Platelet-Rich Fibrin approached significance (*p* = 0.030).

All three groups exhibited a significant increase in gingival thickness from baseline to 6 months. The Connective Tissue Graft increased from a median of 1.00 mm to 2.15 mm (*p* = 0.012), the Fascial Lata Allograft from 1.00 mm to 1.80 mm (*p* = 0.011), and the Platelet-Rich Fibrin from 1.00 mm to 1.55 mm (*p* = 0.011). Among the groups, the Connective Tissue Graft showed the largest gain in gingival thickness, highlighting its superior effectiveness in enhancing gingival tissue thickness.

Table (2) showed At baseline, the CTG group demonstrated a slightly higher mean labial bone thickness compared to the other groups (1.763 ± 0.160 mm) compared to the Fascia Lata Allograft (1.600 ± 0.120 mm) and Platelet-Rich Fibrin (1.613 ± 0.164 mm) groups; however, this difference was not statistically significant (F = 2.939, *p* = 0.075). After 6 months, the mean labial bone thickness increased in all groups, with the highest value observed in the Connective Tissue Graft group (2.300 ± 0.131 mm), followed by Fascial Lata Allograft (2.238 ± 0.119 mm) and Platelet-Rich Fibrin (2.150 ± 0.151 mm), though the overall between-group difference was no longer statistically significant (F = 2.518, *p* = 0.105).

Also, within group changes from baseline to 6 months, all groups showed an increase in labial bone thickness. The Connective Tissue Graft group demonstrated the largest gain, from 1.763 mm to 2.300 mm (p = 0.032), followed by the Platelet-Rich Fibrin group (1.613 mm to 2.150 mm, p = 0.044) and the Fascial Lata Allograft group (1.600 mm to 2.238 mm, p = 0.323). Representative CBCT cross-sectional images illustrating labial bone thickness measurements at baseline and 6 months are shown in Fig. 6(CTG group) and Fig. 7 (FLA group).”

In summary, at 6 months all three approaches achieved significant improvements in the peri-implant soft tissue phenotype, with CTG > FLA > PRF in magnitude of thickening. FLA allograft closely approximated CTG in many parameters without a second surgical site, while PRF provided more modest gains but excellent tissue health. All methods maintained peri-implant bone stability and health over the observation period.

No adverse events observed.

All randomized participants were included in the analysis and analyzed according to their allocated groups. No missing data were observed during the study period; therefore, no imputation methods were required.

## Discussion

The present randomized clinical trial evaluated the comparative effectiveness of three soft tissue augmentation approaches—subepithelial connective tissue graft (CTG), fascia lata allograft (FLA), and platelet-rich fibrin (PRF)—in modifying the peri-implant soft tissue phenotype in patients with thin biotype. Within the limitations of this 6-month follow-up, all three interventions resulted in measurable improvements in gingival thickness, keratinized tissue width, and labial bone thickness. However, the magnitude and clinical impact of these changes varied among the modalities.

Autogenous CTG remains the gold-standard for peri‐implant soft tissue augmentation. CTG reliably increases gingival thickness and keratinized mucosa width, which helps stabilize the peri‐implant seal and supports esthetic outcomes. However, CTG requires a second surgical site (palate or tuberosity) and is limited by donor‐tissue availability and patient morbidity. To overcome these drawbacks, less invasive alternatives – such as xenogeneic collagen matrices, platelet‐rich fibrin (PRF), and allogeneic fascia lata allografts (FLA) – have been proposed. These graft substitutes act as scaffolds rich in growth factors or collagen, enabling augmentation without palatal harvesting [20].

Our trial showed that CTG produced the largest increases in mucosal thickness and Keratinized tissue width. In support of this, 1-year RCT done by Cosyn et al. demonstrated that the increase in buccal soft tissue thickness at 1 year with the mean difference of 0.41 mm in favor of CTG was significant (*p* < 0.001) [21]. In addition, Zucchelli et al. reported that CTG group show more increase in keratinized tissue width 4.27(± 1.16) than PRF group 3.67(± 1.21) [14].

Also, our trial reported that there were increase in median of gingival thickness within PRF group from 1.00 mm (baseline) to 1.55 mm (post 6 month). In agreement of our result, there is split mouth randomized clinical trial done by Patnaik and colleagues, showed that a statistically significant increase in soft tissue thickness at 4 weeks (3.73 ± 0.34 mm vs. 2.21 ± 0.37 mm) and 6 weeks (4.20 ± 0.37 mm vs. 2.4 ± 0.48 mm) at the buccal site for the test group [22] .

Additionally, systematic reviews of randomized clinical trials by Giammarinaro et al., which reported that although PRF significantly improves peri-implant soft tissue thickness, CTG generally achieved greater tissue thickening because of its structural connective tissue characteristics [12].

Despite its clinical effectiveness, CTG is associated with notable limitations, including donor site morbidity, increased surgical time, and limited tissue availability. These drawbacks have driven the development and evaluation of alternative biomaterials, such as FLA and PRF, which aim to achieve comparable outcomes with reduced invasiveness.

Platelet-rich fibrin (PRF) provided moderate augmentation in our study. This is consistent with A 3-months randomized trial carried out by Ustaoglu et al. comparing PRF vs. CTG and revealed that increased keratinized tissue width more among CTG 3.83 ± 0.91 than among PRF 3.21 ± 0.47 with statically significant difference between groups. Furthermore, there is significant increase of keratinized tissue width at 3months postoperatively compared with the baseline values in both groups approximately by 0.09 mm within PRF group and by 0.27 mm within CTG group. These autogenous materials play a role similar to that of a space maintainer. The histoconduction effects of these materials can explain the increased tissue thickness [23].

Importantly, PRF’s biological advantages and ease of use – it is prepared autologously from blood and applied without a second incision – translate to lower cost and morbidity. In practice, PRF can accelerate wound healing and supplement graft volume, making it a clinically attractive minimally invasive option especially for smaller defects, even if its volumetric gains may not reach CTG levels [24].

Moreover, there is trial done by El-Sayed and Hashem reported that the mean ± SD labial bone thickness 6 months postoperative was lower in PRF group (1.1 ± 0.37) than the control group 1.2 ± 0.41). It is concluded that PRF implants have some benefits in preservation of alveolar bone integrity and improving the esthetic outcome in immediate implant [25]. Chung et al. stated that studies have provided evidence that the amount of keratinized mucosa width, which denotes the height of keratinized soft tissue that runs apicocoronally from the mucosal margin to the mucogingival junction, determines the amount of soft-tissue recession and extent of bone resorption after implant placement [18].

Fascia lata allograft (FLA) is a new soft tissue substitute with promising properties. The fascia lata allograft (FLA) demonstrated promising results in the present study, with gingival thickness and labial bone thickness approaching those achieved by CTG.

In the present study, FLA demonstrated favorable integration into the peri-implant mucosa. Compared to palatal connective tissue grafts, fascia lata grafts exhibit reduced postoperative morbidity by limiting surgical intervention to a single site. Furthermore, Histological studies have demonstrated that fascia lata allografts integrate with host tissues through fibroblast infiltration and vascularization, supporting their role in soft tissue augmentation. Given these attributes, fascia lata represents a promising candidate for advancing periodontal regenerative therapies [16].

It is well recognized that CBCT measurements of thin cortical bone have inherent limitations in accuracy. When the labial bone plate thickness is less than 1 mm, partial-volume averaging artifacts and the limited spatial resolution of CBCT may result in overestimation or underestimation of the true bone thickness. Therefore, CBCT measurements of facial bone thinner than 1 mm should be interpreted with caution. In the present study, all baseline labial bone thickness (LBT) measurements exceeded 1 mm (FLA: 1.600 ± 0.120 mm; PRF: 1.613 ± 0.164 mm; CTG: 1.763 ± 0.160 mm), which reduces—although does not completely eliminate—the impact of this limitation on our baseline and 6-month measurements [18].

We acknowledge that soft tissue changes were assessed solely by linear transgingival probing, which, while clinically standard and reproducible, does not capture three-dimensional volumetric alterations. Contemporary digital workflows employing intraoral scanning with model registration and superimposition can provide precise quantification of total soft tissue volume gain. The absence of such volumetric analysis in the present study represents a methodological limitation. Future investigations should incorporate 3D digital assessment methods to complement linear measurements and provide a more comprehensive evaluation of soft tissue augmentation outcomes [26].

An important consideration when interpreting the present findings is the different resorption kinetics of the graft materials. Compared with connective tissue grafts, fascia lata allografts undergo more rapid early resorption, which may partly explain the slightly lower tissue thickness observed in the FLA group at 6 months. In contrast, PRF membranes are resorbed within approximately 2–3 weeks; therefore, the soft tissue changes observed at 6 months are more likely attributable to the biological effects of released growth factors and host tissue remodeling than to the persistence of the PRF matrix itself [14, 27].

### Limitation

The present study has several limitations, including the relatively small sample size and short follow-up period, which may limit the generalizability of the findings. In addition, soft tissue changes were assessed using linear transgingival probing rather than three-dimensional volumetric analysis, and formal examiner calibration and intra- or inter-examiner reliability assessments were not performed. Although all measurements were obtained by a single experienced blinded examiner using a standardized protocol, transgingival probing remains susceptible to operator-dependent variability and soft tissue compressibility. Future studies should include larger cohorts, longer follow-up periods, examiner calibration, and digital three-dimensional assessment techniques to provide more comprehensive and reproducible evaluations of peri-implant soft tissue augmentation.

## Conclusion

Within the limitations of this 6-month RCT, we conclude that all three soft tissue augmentation techniques fascia lata allograft, platelet-rich fibrin, and subepithelial connective tissue graft – were effective in enhancing the peri-implant soft tissue phenotype around anterior implants with initially thin biotypes.

The CTG remains the gold standard for achieving maximum thickness, the FLA emerged as a highly promising, minimally invasive substitute. The FLA achieved tissue gains and aesthetic outcomes nearly comparable to the CTG without the need for a secondary palatal donor site.

In contrast, PRF provided more modest volume improvements, making it less ideal for major phenotype modifications. However, PRF significantly enhanced early wound healing and remains a viable option for minor enhancements or when traditional grafting is contraindicated.

Despite differences between techniques, all approaches maintained peri-implant tissue stability and minimal bone loss over the study period. Ultimately, augmenting thin tissue is clinically vital for long-term stability, with the FLA offering an excellent balance between surgical efficacy and patient comfort.

### Clinical relevance

Augmenting thin peri-implant soft tissues is beneficial for aesthetic and biological outcomes. While the connective tissue graft remains the gold standard for thickening peri-implant mucosa, the use of a fascia lata allograft can achieve comparable soft tissue improvements without donor site morbidity. Platelet-rich fibrin, though less effective in volume augmentation, enhances soft tissue health and may be used when only a minor gain is needed or in conjunction with other grafts. Clinicians now have multiple evidence-based options to optimize the peri-implant tissue phenotype, thereby improving implant prognosis in the esthetic zone.

## Data Availability

The data that support the findings of the current study are available from corresponding author upon resonable request.
